# Altered plasma metabolites and inflammatory networks in HIV-1 infected patients with different immunological responses after long-term antiretroviral therapy

**DOI:** 10.3389/fimmu.2023.1254155

**Published:** 2023-09-27

**Authors:** Lianfeng Lu, Yang Yang, Zhangong Yang, Yuanni Wu, Xiaosheng Liu, Xiaodi Li, Ling Chen, Yang Han, Xiaojing Song, Ziqing Kong, Wei Cao, Taisheng Li

**Affiliations:** ^1^Department of Infectious Diseases, Peking Union Medical College Hospital, Peking Union Medical College and Chinese Academy of Medical Sciences, Beijing, China; ^2^Calibra Lab at DIAN Diagnostics, Hangzhou, China; ^3^Key Laboratory of Digital Technology in Medical Diagnostics of Zhejiang Provinces, Hangzhou, China; ^4^School of Medicine, Tsinghua University, Beijing, China; ^5^State Key Laboratory of Complex Severe and Rare Diseases, Peking Union Medical College Hospital, Chinese Academy of Medical Science and Peking Union Medical College, Beijing, China

**Keywords:** HIV, metabolomics, ART, immune reconstitution, inflammation

## Abstract

**Background:**

Chronic metabolic changes relevant to human immunodeficiency virus type 1 (HIV-1) infection and in response to antiretroviral therapy (ART) remain undetermined. Moreover, links between metabolic dysfunction caused by HIV and immunological inflammation in long-term treated individuals have been poorly studied.

**Methods:**

Untargeted metabolomics and inflammatory cytokine levels were assessed in 47 HIV-infected individuals including 22 immunological responders (IRs) and 25 non-responders (INRs) before and after ART. The IRs and INRs were matched by age, gender, baseline viral load, and baseline CD4+T cell counts. Another 25 age-matched uninfected healthy individuals were also included as controls.

**Results:**

Among the 770 plasma compounds detected in the current study, significant changes were identified in lipids, nucleotides, and biogenic amino acids between HIV-infected patients and healthy controls. Principal Component Analysis (PCA) and the Random Forest (RF) model suggested that levels of selected metabolites could differentiate HIV-infected patients clearly from healthy controls. However, the metabolite profiles identified in our patients were similar, and only three metabolites, maltotetraose, N, N-dimethyl-5-aminovalerate, and decadienedioic acid (C10:2-DC), were different between IRs and INRs following long-term ART. The pathway enrichment analysis results revealed that disturbances in pyrimidine metabolism, sphingolipid metabolism, and purine metabolism after HIV infection and these changes did not recover to normal levels in healthy controls even with suppressive ART. Correlation analysis of the metabolism-immune network indicated that interleukin (IL)-10, D-dimer, vascular cell adhesion molecule-1 (VCAM-1), intercellular cell adhesion molecule-1 (ICAM-1), and TNF-RII were positively correlated with most of the significantly changed lipid and amino acid metabolites but negatively correlated with metabolites in nucleotide metabolism.

**Conclusions:**

Significant changes in many metabolites were observed in HIV-infected individuals before and after ART regardless of their immunological recovery status. The disturbed metabolic profiles of lipids and nucleotides in HIV infection did not recover to normal levels even after long-term ART. These changes are correlated with modified cytokines and biomarkers of chronic non-AIDS events, warranting tryout of interventions other than ART.

## 1 Introduction

Although the introduction of antiretroviral therapy (ART) has achieved viral suppression and subsequent immune reconstitution in most people living with HIV-1 (PLWH), increased risks of chronic AIDS-related illnesses associated with aging are still challenging in this population (1). Nevertheless, 10-30% of PLWH suffer from poor immune recovery despite prolonged successful suppressive ART, which further speeds up the disease progression and contributes to increased mortality, referred to as “immunological non-responders” (INRs) (1, 2). Though there was no unified standard for INRs, the CD4+T cell count could not reach 350 cells/μl or 500 cells/μl after years of ART was used in some reports (3, 4). Growing evidence has revealed that HIV infection could induce various metabolic changes including impaired glucose metabolism (5, 6), hypertriglyceridemia (5), downregulation of sphingomyelin metabolism (6), and so on. These changes are related to the effects of HIV infection on the proliferation and functioning of immune cells. For example, the transition of resting T cells depends on the catabolic metabolism of glucose and the displacements of amino acids and fatty acids in oxidative phosphorylation; susceptibility of CD4+T cells to HIV-1 causes increased glycolysis and oxidative phosphorylation (OXPHOS) independent of the activation phenotype (7). However, current results of HIV-caused metabolic changes are not consistent, and metabolic changes in HIV patients after long-term ART are unclear.

Metabolomics has been widely applied to screening potential biomarkers and related disease mechanisms, which has been applied to liver disease (8), cardiovascular disease (9), cancer (10), and HIV infection (6, 7, 11, 12). Several metabolomics studies of HIV infection suggest that ART could only partially recover the disturbed metabolic changes due to HIV infection (11, 13). Some antiretroviral drugs may even cause additional metabolic dysfunction (7, 14), such as hyperactive glycolysis (13), enhanced mitochondrial toxicity (13), abnormal amino acid catabolism (11, 12, 15), and imbalances in phospholipid and sphingolipid metabolism (11, 12). While two studies have reported the effects of long-term ART on HIV patients’ metabolism (12, 16), little has been explored regarding metabolic changes between INRs and IRs of ART. Higher accumulation of plasma acylcarnitine (17) and persistent decline in sphingosine-1-phosphate phosphatase 1 activity have been observed in INRs (6), while more high-density lipoprotein (HDL) particles, HDL cholesterol, and microbial translocation have been reported in IRs (16, 18). However, the available study results regarding IRs and INRs were not consistent.

Therefore, we plan to comprehensively assess the immune-metabolic adaptations occurring with HIV infection and the potential response to long-term ART. The plasma metabolomes were studied in HIV-infected individuals before and following long-term ART according to their varied immune responses and compared with HIV-seronegative people. The study results provide clues for developing improved treatment strategies for the well-being of HIV patients.

## 2 Materials and methods

### 2.1 Study subjects and study design

**Figure 1:**
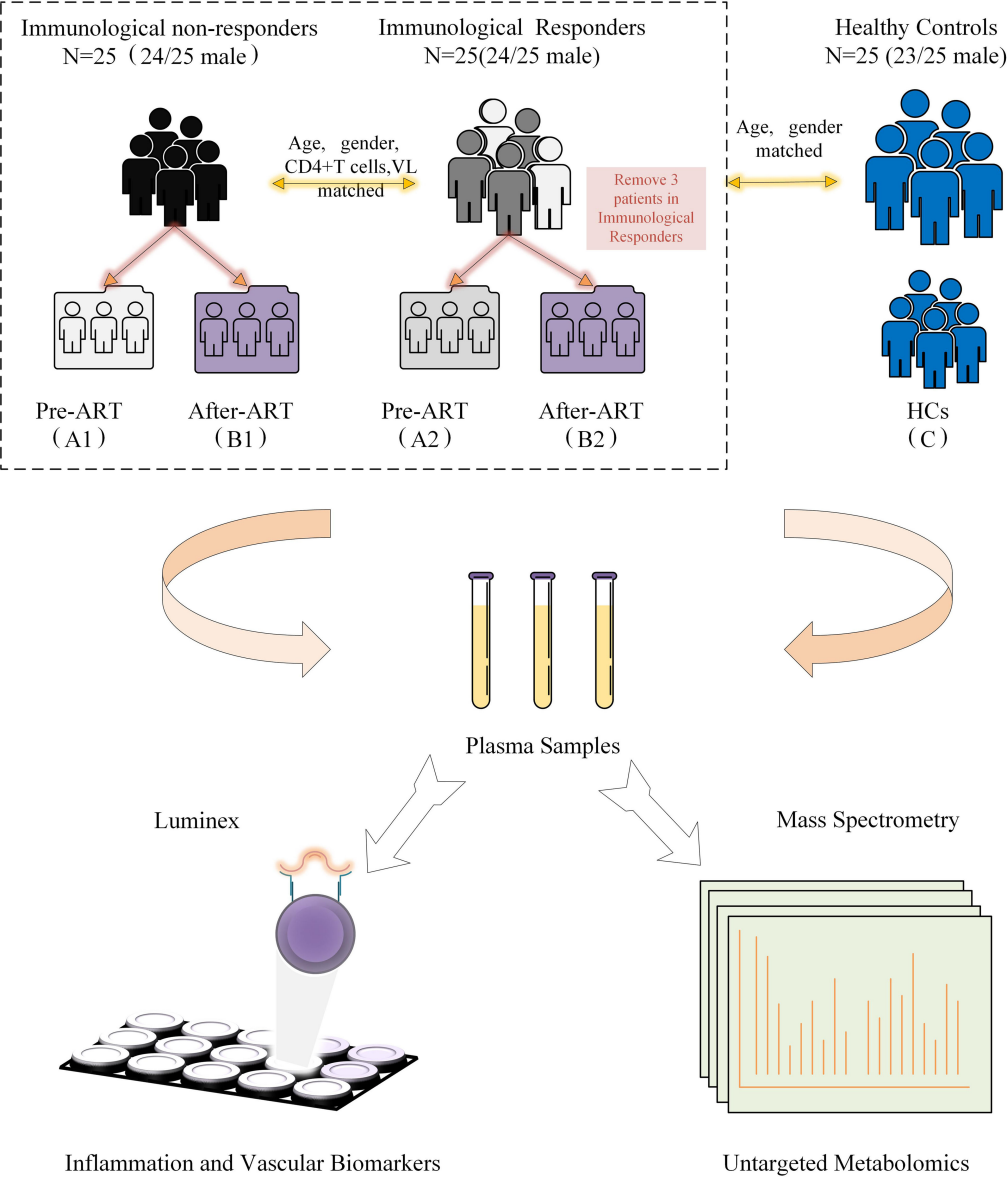
Diagrammatic flow chart of study design. VL, Viral load; HCs, Healthy controls; ART, Antiretroviral treatment.

Eligible participants were recruited from the HIV/AIDS outpatient clinic, Peking Union Medical College Hospital (PUMCH), China. All participants have been regularly followed with suppressed viremia (<50 copies/ml) for at least 3 years. Our definition of INRs (n=25, male 24/25) was identified as ART>3 years with viral suppression and CD4+T cell ≤ 350 cells/μl. IRs (n=25, male 24/25) were identified as ART>3 years with viral suppression and CD4+T cell≥500 cells/μl (**Figure 1**). HIV-seronegative subjects matched by age and gender (n=25, male 23/25) to the HIV patients were recruited at the same time as controls. The IRs and INRs were matched by baseline CD4+T cells, viral loads, age, and gender, and HIV patients co-infected with HBV or HCV were excluded. Demographics and clinical data of the participants were collected from this mini study. Samples from different time points were analyzed according to the study protocol. The study was approved by an independent ethics committee and the institutional review board of PUMCH (Peking Union Medical College Hospital, JS-1431).

### 2.2 Laboratory testing

#### 2.2.1 Untargeted metabolomics

Untargeted metabolomic analysis was conducted by Calibra Lab at DIAN Diagnostics (Hangzhou, Zhejiang, China) on their CalOmics metabolomics platform. Samples were extracted using methanol in a ratio of 1:4. The mixtures were shaken for 3 minutes and precipitated by centrifugation at 4000 × g, 10 minutes at 20 °C. Four aliquots of 100 μL supernatant were transferred to sample plates and dried under blowing nitrogen, then re-dissolved in reconstitution solutions for sample injection into UPLC-MS/MS systems. The instruments for the four UPLC-MS/MS methods are ACQUITY 2D UPLC (Waters, Milford, MA, USA) plus Q Exactive (QE) hybrid Quadrupole-Orbitrap mass spectrometer (Thermo Fisher Scientific, San Jose, USA). QE mass spectrometer was operated at a mass resolution of 35000, the scan range was 70-1000 m/z. In the first UPLC-MS/MS method, QE was operated in positive ESI mode and the UPLC column was C18 reverse-phase (UPLC BEH C18, 2.1x100 mm, 1.7 um; Waters); the mobile solutions used in the gradient elution were water (A) and methanol (B) containing 0.05% PFPA and 0.1% FA. In the second UPLC-MS/MS method, QE was operated in negative ESI mode, and the UPLC column was C18 reverse-phase (UPLC BEH C18, 2.1x100 mm, 1.7 um; Waters), the mobile solutions used in the gradient elution were water (A) and methanol (B) containing 6.5 mM ammonium bicarbonate at pH 8. The third UPLC-MS/MS method had the QE operated in ESI positive mode and the UPLC column was C18 reverse-phase (UPLC BEH C18, 2.1x100 mm, 1.7 um; Waters), the mobile solutions were water (A) and methanol/acetonitrile/water (B) contain 0.05% PFPA and 0.01% FA. In the fourth method, QE was operated in the negative ESI mode, the UPLC column was HILIC (UPLC BEH Amide, 2.1x150 mm, 1.7 um; Waters), and the mobile solutions were water (A) and acetonitrile (B) with 10 mM ammonium formatted.

After raw data pre-processing, peak finding/alignment, and peak annotation using in-house software, metabolites were identified by searching an in-house library containing more than 3,300 standards with library data entries generated from running purified compound standards through the experimental platforms. Identification of metabolites must meet three strict criteria: narrow window retention index (RI), accurate mass with variation less than 10 ppm, and MS/MS spectra with high forward and reverse scores based on comparisons of the ions present in the experimental spectrum to the ions present in the library spectrum entries. Almost all isomers can be distinguished by these three criteria. All identified metabolites meet the level 1 requirements by the Chemical Analysis Working Group (CAWG) of the Metabolomics Standards Initiative (MSI) except some asterisk-labeled lipids in which MS/MS spectral were in silico matched. Based on this metabolomics platform, many peer-reviewed articles have been published (19–21).

### 2.3 Virological and immunological measurements

The plasma HIV-1 RNA load was measured using the COBAS Ampliprep/TaqMan 48 real-time RT-PCR Test (Roche, CA, USA) according to the manufacturer’s instructions. Immune profiles of peripheral blood lymphocytes were analyzed by three-color flow cytometry (Epics XL flow cytometry; Beckman Coulter, USA) as previously described (22). The inflammatory cytokine panel was measured by a standardized Luminex assay [(Bio-Rad Laboratories, Hercules, CA, USA)] according to the manufacturer’s instructions, and 14 cytokines were measured: interleukin (IL)-1β, IL-2, IL-6, tumor necrosis factor (TNF)-RII, IL-8, IL-10, IL-7, Chemokineligand-10 (CXCL-10), Monocyte chemotactic protein-1 (MCP-1), Vascular Cell Adhesion Molecule-1 (VCAM-1), Intercellular Cell Adhesion Molecule-1 (ICAM-1), D-dimmer, interferon (IFN) -γ, and soluble CD40 ligand (CD40L).

### 2.4 Statistical analysis

The statistical analysis and figure drawing were conducted using Metaboanalyst 5.0 software (www.metaboanalyst.ca/), Originpro 2023 software, and R statistical package version 4.0.0 (R Foundation for Statistical Computing, Vienna, Austria). For the descriptive comparisons, categorical variables were compared with the chi-square test and continuous variables with student t-test. For the metabolomics data, raw peak areas were median normalized to adjust for system fluctuation among different run days. The normalized peak areas were log-transformed to reduce data distribution bias and to be in an approximate normal distribution (Gaussian distribution). Missing values in the peak matrix (under detection limit) were imputed with the minimum detection value of a metabolite in all samples. Unsupervised Principal Component Analysis (PCA) and supervised Partial Least Square Discrimination Analysis (PLS-DA) were conducted to visualize sample clustering. Besides, PLS-DA was used to pick up the variable importance in the projection (VIP) values in the first principal component (PC), as part of the principle of metabolite selection (15, 23). Based on their VIP values of PLS-DA analysis, metabolites with VIP >1.0 and p <0. 05 in the Welch’s t-test and volcano plots were selected as the differentially expressed metabolites. After the Benjamin–Hochberg procedure was applied to control the false discovery rate (FDR) due to multiple testing, metabolites with log 2 (fold change) >1 were finally selected as significantly changed metabolites. The above statistical criteria have been designed to select mainly biologically relevant metabolites and avoid nonspecific noisy signals in the study.

Result visualizations were provided for the performed statistical analyses, including volcano plots in differential metabolite tests (which were performed in Metaboanalyst 5.0 software), scatter plots with confidence ellipse in PCA, scatter plots with a confidence ellipse and variable importance dot plots in PLS-DA, and a variable mean decrease accuracy dot plot in RF.

**Table:** 

Software	Version code	Website
R	3.4.1	http://www.cran.com
Rstudio	1.4.1717	https://www.rstudio.com
mixOmics	6.10.9	http://mixomics.org/
randomForest	4.6-14	https://www.rdocumentation.org/packages/randomForest/versions/4.6-14

The differentially expressed metabolites between groups were mapped into biochemical pathways, and the major disturbed metabolic pathways were compared between groups by Holm-Bonferroni adjusted p value. Pathways with p <0.1 were considered significantly modified and further investigated. Heat maps and correlation matrix were generated for visualization. Spearman correlations were conducted to study the associations among deferentially expressed metabolites, clinical data, and cytokine data.

## 3 Results

### 3.1 Demographic characteristics

The metabolomic features were studied in three types of participants, including group 1 of HIV-infected INRs (n=25), group 2 of HIV-infected IRs (n=25), and group C of healthy controls (HCs). In HIV-infected groups 1 and 2, sequential samples were evaluated including a pre-ART baseline point (A, groups A1 and A2) and a post-ART point (B, groups B1 and B2). In addition, fasting blood from HIV seronegative subjects (n = 25) was obtained in a single visit (Flow chart in **Figure 1** and characteristic of patients seen in **Table S1**). However, verification of clinical information revealed that three subjects developed lymphoma or used other adjuvant agents to improve CD4 count in follow-up. Therefore, their associated metabolite information and cytokines data were removed after follow-up analysis. All participants were balanced among age, gender, as well as the baseline viral load and CD4+T cell counts (**Table 1**). Besides, the total cholesterol, blood glucose level, proportion of high blood pressure, and ART regimens were also similar during follow-up, which ensured the equivalent possibility of other comorbidities (**Table 1**). Overall, the average time of treatment and follow-up in all HIV-1-infected patients was 6.81 ± 3. 10 years. Clinical and demographic characteristics were also balanced among groups (see in **Table 1**).

**Table 1:** Clinical characteristics of patients.

Characteristics	INRs	IRs	HCs	p-value
	CD4≤350	CD4 ≥ 500	N=25	
	N=25	N=22		
Age, mean years (SD)	45.8±11.7	45.9±11.0	44.7±12.4	0.925
Male, n (%)	24 (96.0)	21 (95.4)	23 (92.0)	0.801
Route of transmission, n (%)				
Sexual				
Blood	20 (80)	17 (77.3)		
Unclear/others	2 (8.0)	2 (9.1)	–	0.974
	3 (12.0)	3 (13.6)		
Initial ART regimen, (%)				
2 NRTIs + NNRTI	20 (80.0)	18 (81.8)	–	0.384
2NRTIs+PI	1 (4.0)	2 (9.2)		
2 NRTIs + INSTI	4 (16.0)	1 (4.5)		
Other	0 (0.0)	1 (4.5)		
Switching ART regimen, n (%)				
2 NRTIs + NNRTI				
2NRTIs+PI	17 (68.0)	12 (54.5)	–	0.473
2 NRTIs + INSTI	4 (16.0)	4 (18.2)		
Other	4 (16.0)	4 (18.2)		
	0 (0.0)	2 (9.1)		
Viral load (lg copies/mL)	5.07±0.73	5.22±0.71	–	0.658
Weight(kg)	59.8±10.7	65.4±10.3	–	0.107
Blood Glucose (mmol/L)	5.09±0.49	5.07±0.91	5.46±0.70	0.098
Total Cholesterol (mmol/L)	4.35(3.45,5.07)	3.99(3.38,4.72)	4.94(4.53,5.64)	0.002*
High Blood Pressure, n (%)	1(4.8)	3(14.3)	–	0.606
Blood Glucose after ART (mmol/L)	5.30(4.95,5.85)	5.30(5.00,5.75)	–	0.771
Total Cholesterol after ART (mmol/L)	4.23(3.83,5.40)	4.82(4.29,5.12)	–	0.125
Baseline CD3+CD4+T counts (cells/μl )	24 (5, 63)	41 (11, 52)	–	0.572
Peak CD3+CD4+T counts (cells/μl )	244 (201,292)	630 (540,788)		<0.001

*significant p-value of INRs with HCs and IRs with HCs by Bonferroni correction; p-value <0.05; INRs, Immunological non-responders; IRs, Immunological responders; HCs, Healthy controls; SD, Standard deviation; NRTIs, Nucleoside reverse-transcriptase inhibitors; NNRTIs, Non-Nucleoside reverse-transcriptase inhibitors; PI, Proteinase inhibitor; INSTIs, Integrase inhibitor, -, no relavent data.

### 3.2 Altered plasma metabolite summary

#### 3.2.1 PCA and PLS-DA analysis

**Figure 2:**
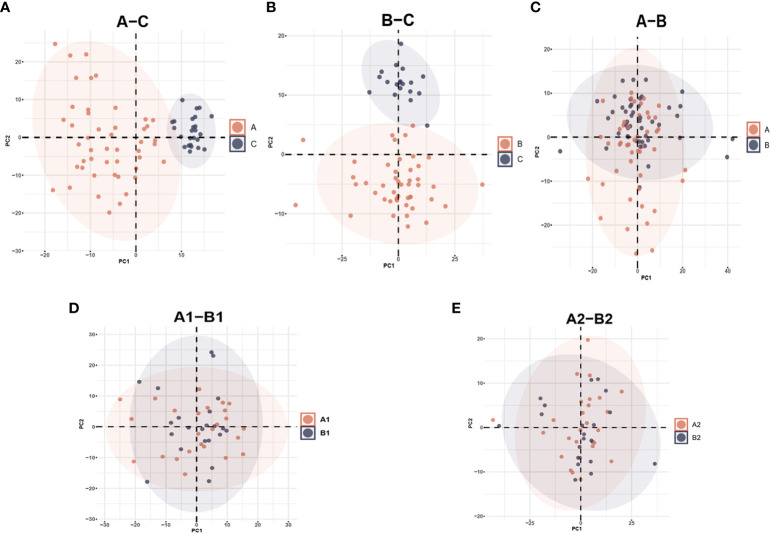
Principal Component Analysis (PCA) among different groups comparison: **(A)** PCA plot identified group of HIV-infected patients prior to antiretroviral treatment (A) and healthy controls (C); **(B)** HIV-infected patients after antiretroviral treatment **(B)** and healthy controls (C); **(C)** HIV-infected patients prior to antiretroviral treatment (A) and after antiretroviral treatment (B); **(D)** HIV immunological non-responders (A1) and immunological responders (A2) prior to antiretroviral treatment; **(E)** immunological non-responders (B1) and immunological responders (B2) after antiretroviral treatment.

From the original list of detected metabolites in the metabolomics analysis, 770 out of 938 metabolites were selected that are endogenous and with a confirmed identity. The 770 metabolites could be divided into six categories: lipids, amino acids, carbohydrates, nucleotides, cofactors and vitamins, and peptides. PCA and PLS-DA models were performed to determine variation between groups. In the PCA model, a clear mode of separation between PLWH and HCs was found; there was more centralized in HCs and more scattering in HIV patients (**Figures 2A, B**, A–C, B–C). However, there is some overlapping of PLWH seen in a pre-treatment and after-treatment comparison, and the differences between INRs and IRs before and after ART in PCA analyses were less obvious (**Figures 2C–E**). A similar separation between the groups was also observed in the PLS-DA analyses (**Figure S1**, **Supplementary Materials**).

#### 3.2.2 Significant metabolites summary

The Welch’s t-test and PLS-DA method identified significantly different metabolite levels between groups. When comparing untreated HIV patients with the healthy controls, 55 metabolites were upregulated and 30 were downregulated. Of the 55 upregulated metabolites, 60% were lipid metabolites and 16% were amino acid metabolites. Of the 30 downregulated metabolites, 68% were lipid metabolites and 18% were amino acid metabolites (**Figure S2A** and **Table S2**). After long-term ART, HIV patients had 50 significantly higher and 28 lower levels of metabolites compared with the HCs. In the comparisons between pre-ART and after-ART HIV patients (A–B), after-ART HIV patients had 7 downregulated metabolites and 31 upregulated metabolites. Two of the seven downregulated metabolites were 2’-deoxyuridine and N-carbamoylaspartate, and the rest were lipids (**Figure S2B** and **Table S2**). Of the significantly upregulated metabolites in the HIV-infected patients compared with healthy controls (B–C), 60% were lipid, 16% were amino acids and 10% were nucleotide (**Figure S2C** and **Table S2**).

**Figure 3:**
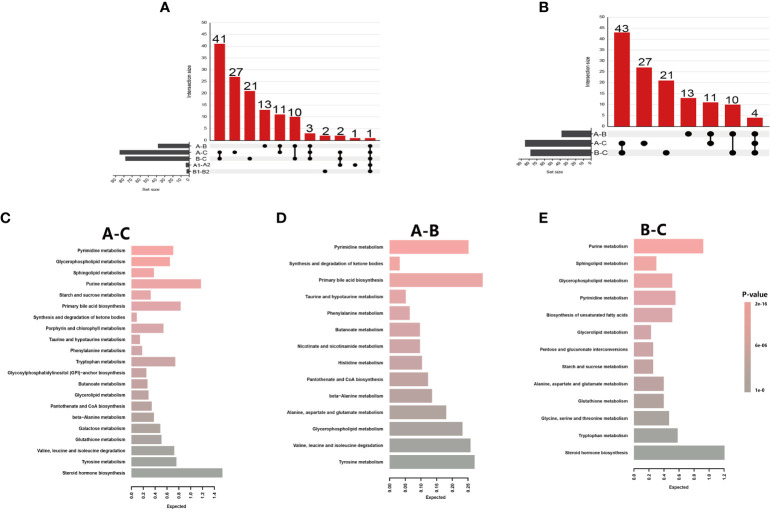
Metabolite summaries and pathway enrichment analyses of different groups. **(A)** Upset figure of five groups of comparisons (A-C, A-B, B-C, A1-B1, A2-B2) plotting the intersections of a set as a matrix. Each column corresponds to a set, and the bar charts on top show the size of the set. Each row corresponds to a possible intersection: the filled-in cells show which set is part of an intersection; **(B)** Upset figure of three groups of comparisons (A-C, A-B, B-C) plotted the intersections of a set as a matrix; **(C)** Metabolite Set Enrichment Analysis using 71 named metabolites which could distinguish HIV patients’ pre-antiretroviral treatment (ART) vs healthy controls (A-C); **(D)** A total of 25 named metabolites which could distinguish HIV patients’ pre-ART vs HIV patients after-ART (A-B); **(E)** A total of 68 named metabolites which could distinguish HIV patients after-ART vs healthy controls (B-C).

To find metabolites with different levels between IRs and INRs, significant metabolites were compared at baseline and post-ART (pre-treatment: A1-A2, after-treatment: B1-B2, **Figures S2D, S2E**). Four significant metabolites were prominent in the comparison before treatment (A1-A2, **Figure S2D**) and three in the comparison after treatment (B1-B2, **Figure S2D**). Maltotetraose was downgraduated while dihydroorotate and beta-cryptoxanthin were upgraduated in A1 compared with A2. Maltotetraose and N, N-dimethyl-5-aminovalerate were lower while decadienedioic acid (C10:2-DC) was higher in B1 compared with B2. The upset diagrams summarizing metabolite comparisons are shown in **Figures 3A, B**. Maltotetraose, which was a glucose residue at the reducing end, was the only metabolite regulated in all groups at the same time. It remained increased in all HIV-infected patients compared with HCs, particularly prominent before ART initiation. In addition, INRs were associated with lower levels of maltotetraose than IRs no matter whether they received ART or not. Meanwhile, there are other two significant metabolic bio-chemicals, higher N-stearoyl-sphinganine (d18:0/18:0) and higher 1-stearoyl-2-oleoyl-GPS (18:0/18:1), and these are commonly regulated in HIV-infected patients compared with HCs. They were also higher in PLWH before ART compared with those after ART. The 5-hydroxyhexanoate, however, showed upregulation in HIV-infected patients compared with HCs but was downregulated after ART initiation (differential metabolites for each comparison in **Table S2**).

#### 3.2.3 Significant pathway summary

The significantly changed metabolites between different groups were applied to pathway enrichment analysis using MetaboAnalyst 5.0 software in the Human Metabolome Database. Since there were only five significantly changed metabolites between INRs and IRs, and the five metabolites belong to five different metabolic pathways, pathway enrichment was not applied to the comparison between INRs and IRs. Further biochemical analysis was mainly targeted on changed metabolites between HIV-infected patients and HCs, as well as between before and after ART HIV patients.

The significantly changed metabolites between different groups were primarily nucleotides, amino acids, and energy metabolism metabolites (**Table S3**). These metabolites between pre-ART HIV patients and HCs belong to 21 different metabolic pathways, and 5 of these 21 pathways had impact>0.1 and p-value<0.05 and were further investigated. The most distinct pathway was glycerophospholipid metabolism with a highest impact value of 0.256, followed by the pyrimidine metabolism, sphingolipid metabolism, purine metabolism, and starch and sucrose metabolism pathway (**Figure 3C**; **Table S3**). Between HIV-infected patients before and after ART, the only enriched was the pathway of synthesis and degradation of ketone bodies with an impact score of 0.6 (**Figure 3D**; **Table S3**). Purine metabolism, sphingolipid metabolism, glycerophospholipid metabolism, and pyrimidine metabolism were from the top down in importance between HIV-infected patients and HCs, with an highest impact score of 0.21 (**Figure 3E**; **Table S3**).

### 3.3 Identification of potential biomarkers

**Figure 4:**
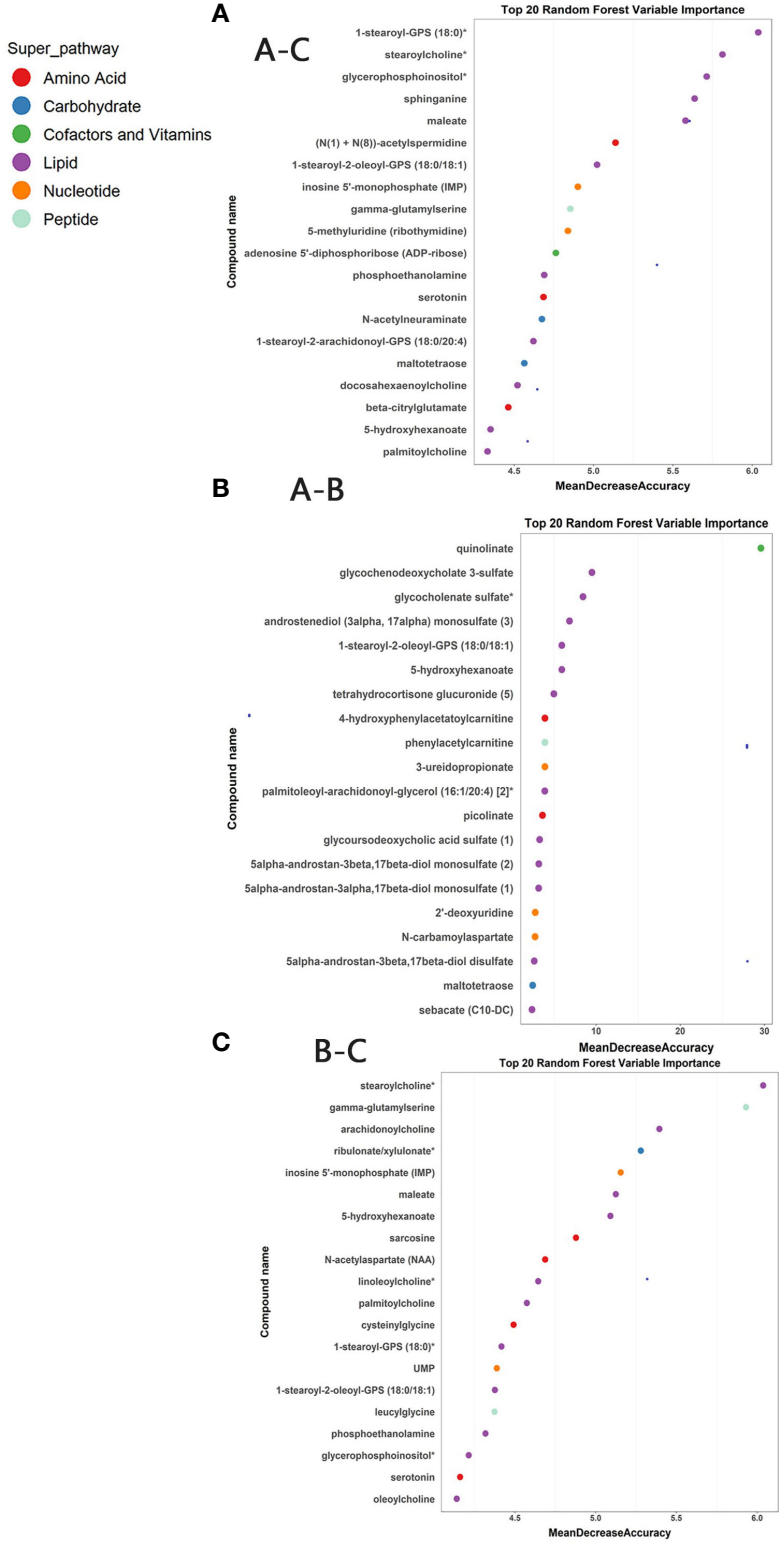
**(A)** Random Forest (RF) analysis of named biochemicals with predictive accuracies of 98.61% for HIV pre-antiretroviral treatment (ART) vs HCs. The biochemical importance plots display the top 20 metabolites which contribute mostly to the groups’ separation based on amino-acid metabolism, lipid metabolism, Nucleotide metabolism, energy metabolism, co-factors, and vitamins and peptides as indicated in different colors in the legend; **(B)** The RF analysis of named biochemicals resulted in predictive accuracies of 87.23% for HIV pre-ART vs. HIV after-ART; **(C)** The RF analysis of named biochemicals resulted in predictive accuracies of 100% for HIV after-ART vs. HCs.

Random forest analyses were conducted to determine potential biomarkers that can differentiate different sample groups. The RF analysis resulted in a predictive accuracy of 98.61% between pre-ART HIV patients and HCs, and a predictive accuracy of 100% between post-ART HIV patients and HCs. For patients infected with HIV before and after ART, the RF analysis had a predictive accuracy of 87.23%. The biochemical importance plots revealed the top 20 metabolites that contributed most to the distinction between baseline PLWH and HCs (**Figure 4A**), between baseline PLWH and post-ART patients (**Figure 4B**), and between post-ART PLWH and HCs (**Figure 4C**). 1-stearoyl-GPS (18:0) was the top metabolite that could be used to distinguish HIV patients from HCs (**Figure 4A**). Quinolinate was the top metabolite to differentiate HIV patients before and after ART (**Figure 4B**), As shown in **Figure 4C**, strearoylcholine contributed most to the separation of post-ART patients and HCs (**Figure 4C**). The top 20 metabolites differentiating group A and C involved in nucleotide metabolism (2/20), amino-acid metabolism (3/20), and lipid metabolism (11/20). Metabolites involved in amino-acid metabolism (4/20) and lipid metabolism (11/20) were among the top 20 metabolites to separate post-ART patients and HCs. Most of the top 20 metabolites in the RF analysis to differentiate pre-ART and post-ART patients were mainly involved in lipid metabolism (12/20). The pathways revealed by the RF analysis were consistent with the pathways identified by the pathway enrichment analysis, pointing to potential biomarkers in lipid, nucleotide, and amino acid metabolisms.

### 3.4 Potential biomarkers correlated with inflammatory factors

The metabolites identified from RF and pathway enrichment analysis as potential biomarkers are further investigated for their correlations with inflammation and cardiovascular disease biomarkers. The levels of different cytokines in plasma are tested to reflect systemic immune activity and disease progression. Thus, the plasma samples tested in the metabolomics analysis were also analyzed for correlation between inflammatory factors and biomarkers for cardiovascular risk. Most of these tested values had significant changes in HIV-infected patients both before and after ART. MCP-1 and CD40L were markedly different between INRs and IRs (**Figure S3**).

**Figure 5:**
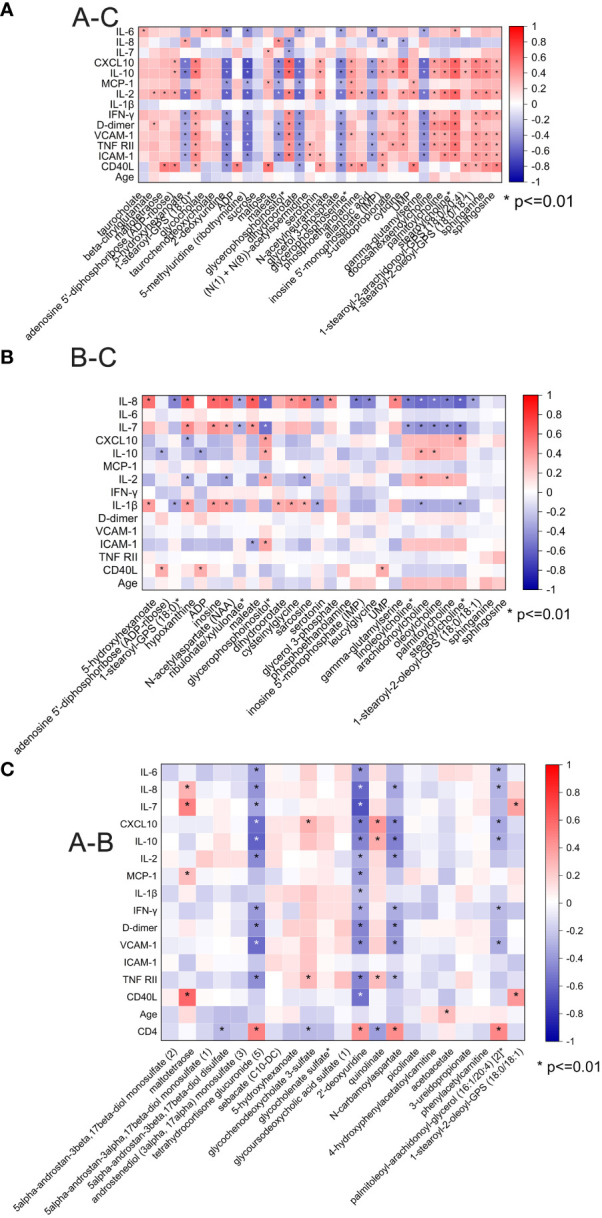
**(A)** Spearman correlation coefficient visualization between metabolites and plasma cytokines in comparison of HIV patients’ pre-antiretroviral treatment (ART) and Healthy Controls (A-C). Metabolites belonging to various categories were plotted in rows and cytokines in columns. The significant level was p <0.01; **(B)** Spearman correlation coefficient visualization in comparison of HIV patients after-ART and HCs (B-C); **(C)** Spearman correlation coefficient visualization in comparison of HIV patients’ pre-ART and after-ART (A-B).

We then analyzed the correlation between the plasma inflammatory factors and changed metabolites between different groups (**Figure 5**). Correlations with a p-value <0.01 were deemed significant. The correlation heatmap of metabolites and inflammatory factors are shown in **Figure 5A** (A–C). Most of the inflammatory factors except for IL-7, IL-8, and IL-1β were correlated with most significantly changed metabolites between pre-ART patients and HCs. Among them, inflammatory factors including CXCL-10, IL-10, MCP-1, D-dimer, VCAM-1, ICAM-1, etc. were negatively correlated with changed nucleotide metabolism metabolites but positively with lipids. However, IL-8, IL-7, and IL-1β were positively correlated with most of the changed acyl cholines after ART (**Figure 5B**). There was no obvious specific characteristic between the plasma cytokines and significantly changed metabolites between pre-ART and post-ART samples (**Figure 5C**). It is of note that two of the top candidates in the RF analysis, N-carbamoylaspartate and 2-deoxyuridine, showed negative correlation with IL-10, IL-8, D-dimer, and VCAM-1, but were positively correlated with CD4+T cells.

### 3.5 Summary of changed metabolites HIV-infected patients before and after ART

**Figure 6:**
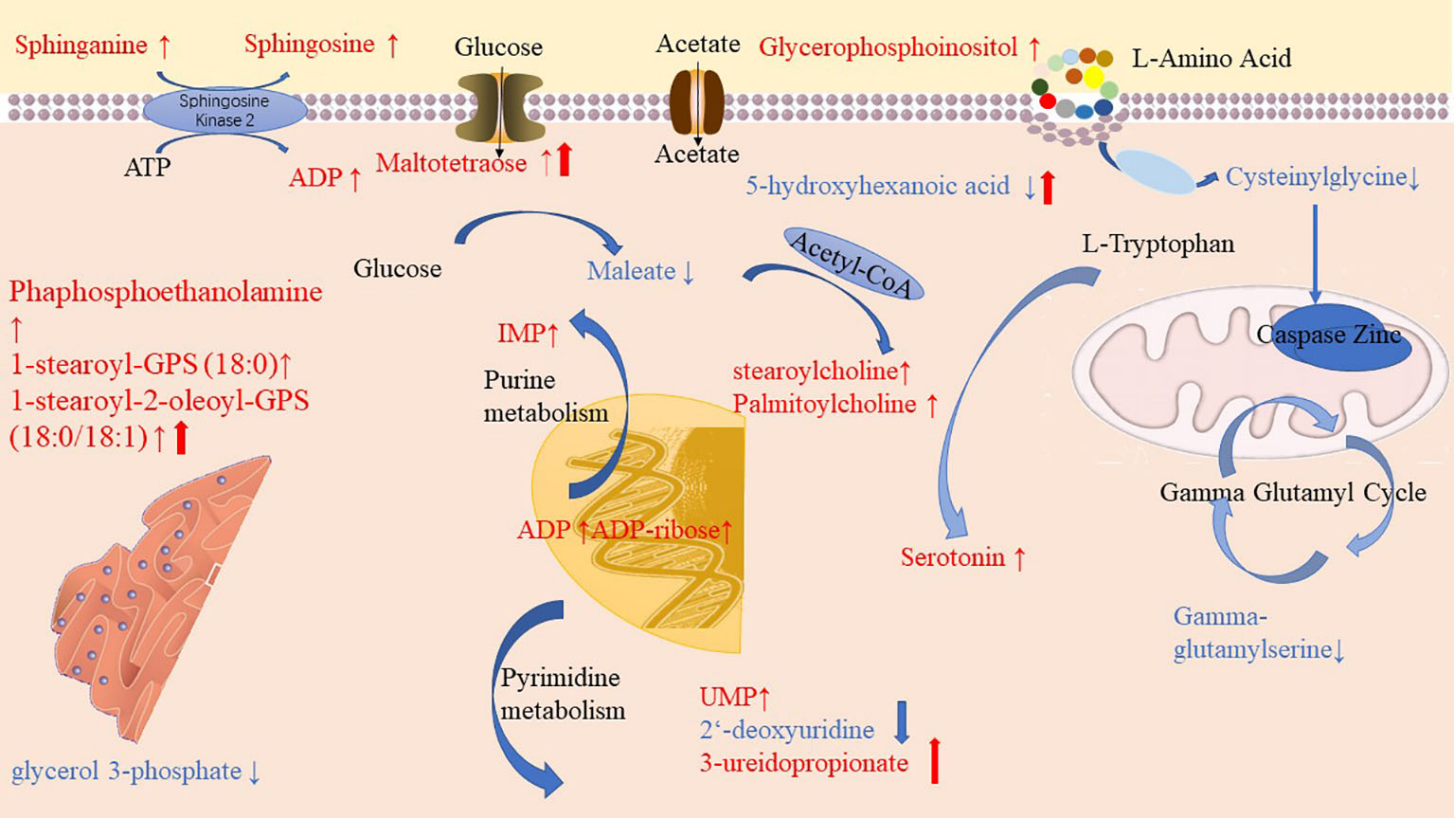
Diagrammatic representation of metabolic pathways modified by HIV and antiretroviral treatment (ART). In pre-ART HIV infected patients, colorful symbols and words in black indicated CoA conjugate, enzyme, and different pathway names. Words in red indicate significantly increased metabolites compared with HCs. Solid-line upwards pointing arrows (↑) indicate significantly increased metabolites in after-ART patients compared with HCs. Words in blue indicates significantly decreased metabolites compared with HCs. Solid-line downwards pointing arrows (↓) indicate significantly decreased metabolites in after-ART patients compared with HCs. The parallel line up-arrows and parallel line down-arrows indicate significantly increased and decreased levels following ART compared with pre-ART, respectively. IMP, inosine 5’-monophosphate; UMP, uridylic acid; ADP, Adenosine diphosphate.

Through pathway enrichment analysis, we found that the levels of many amino acids, lipids, and nucleotides were greatly changed in the PLWH compared with the HCs. The metabolic pathways disturbed by HIV infection and ART were summarized in **Figure 6**. Maltotetraose, metabolite in the glucose metabolism pathway, were higher in pre-ART PLWH than in HCs and further increased after ART. Fatty acid metabolism such as stearoylcholine and palmitoylcholine were significantly higher in both pre-ART and post-ART PLWH than in HCs. The upstream metabolite 5-hydroxyhexanoic acid decreased in HIV-infected patients and further declined after ART. Some phospholipids and lysophospholipids including phosphoethanolamine, 1-stearoyl-GPS (18:0), 1-stearoyl-2-oleoyl-GPS (18:0/18:1), glycerophosphoinositol, sphinganine, and sphingosine were higher in both pre-ART and post-ART PLWH. Although ART significantly decreased the levels of 1-stearoyl-2-oleoyl-GPS (18:0/18:1), it was still higher than in HCs. The only glycerolipid, glycerol 3-phosphate, was lower in PLWH regardless of ART. Some metabolites in purine and pyrimidine metabolism showed variable changes in HIV-infected patients. The pyrimidine metabolites uridylic acid (UMP) and 3-ureidopropionate were higher in PLWH, while 2-deoxyuridine was lower after ART. Metabolites in purine metabolism such as inosine 5’-monophosphate (IMP), adenosine diphosphate (ADP), and ADP-ribose were higher in PLWH than in HCs, and ART treatment had no significant effects on their levels. Finally, serotonin, as the final products of tryptophan and gamma-glutamyl serine, as gamma-glutamyl compounds, demonstrates conversely trends when compared with HC.

## 4 Discussion

Although ART has achieved long-term viral suppression, incomplete immune reconstitution and chronic non-AIDS-related illnesses have always been concerns in PLWH. Since accompanied metabolic changes occurred during HIV infection, whether they could recover after ART treatment and their relationship with immune reconstitution needs further exploration. In this study, we detected 938 metabolites using metabolomics analysis, and further analyzed 770 of them which are endogenous metabolites in PLWH. The omics approach provided an unbiased review of the overall metabolic dynamics. Although significantly different metabolite profiles were revealed between HIV seronegative healthy controls and PLWH with years of ART, metabolic profiles were not markedly affected by different immune reconstitution levels. Collective analysis of metabolomics and inflammatory profiling results further indicated potential metabolic biomarkers identified in PLWH related to immunological factors and cytokines linked to cardiovascular events.

Chronic HIV infection, the resultant inflammation, and ART toxicities could all contribute to activation of immune systems and metabolic imbalance in the host. In line with other publications, our metabolomics results indicated that HIV patients’ metabolic profiles could be clearly separated from HIV seronegative controls (11, 12, 24). The plasma of untreated HIV patients had decreased levels of glutathione and tryptophan pathway metabolites, and increased levels of fatty acids, sphingomyelin, phospholipid, and lysophospholipids compared to HCs (9, 11, 24). Purine and pyrimidine metabolisms in untreated HIV patients were also markedly different from the healthy controls, which were seldom mentioned in previous studies (14, 23, 25). The changed purine and pyrimidine metabolism could be coming from active HIV virion replication that requires high rates of nucleotide turnover. Elevated levels of many acyl cholines in our study might indicate a higher level of β-oxidative stress in PLWH (17, 26). After ART, several metabolites in pyrimidine metabolism including 2-deoxyuridine and 3-ureidopropionate were restored to healthy control levels, while other metabolites such as 1-stearoyl-2-oleoyl-GPS (18:0/18:1), maltotetraose, and 5-hydroxyhexanoate were still different from their levels in healthy controls. Levels of UMP, IMP, ADP, and ADP-ribose were not restored to the healthy control levels. We hypothesized that the underlying biological mechanisms of these differences might be related to ART medication and suppressed HIV virion replication, which needs to be further investigated in future studies.

Previous studies have demonstrated that metabolic changes by HIV infection are associated with host immune profiles and biomarkers of chronic inflammation (7, 11, 27). For instance, N. Chantal Peltenburg et al. reported that sphingomyelins and phospholipids were negatively correlated to Interferon γ-induced protein 10 kDa (IP-10) and sIL-2R, and triglycerides were linked to MCP-1 (11). In our study, we found that IL-10, CXCL10 (IP-10), D-dimer, VCAM-1, TNF-RII, and ICAM-1 were positively correlated with most of the significantly changed metabolites in lipid metabolism from HIV infection, but negatively correlated with changed metabolites in nucleotide metabolism. After ART, most of the changed lipids (mainly acyl cholines) were positively correlated with IL-8, IL-7and IL-1β. It has been reported that accumulation of ICAM-1, TNF-RII, VCAM-1, and D-dimer in the vessel wall is a hallmark of atherosclerosis and acute coronary syndrome and is mediated by the interaction between adhesion molecules on endothelial and circulating cells (28, 29). Proinflammatory cytokine IL-8 (30) and inductive cytokine IL-7 were elevated (31) in HIV infection and promote virus replication (32). The immune-metabolic networks revealed that HIV suppression through ART attenuates the pro-inflammatory status and risk for cardiovascular disease but might contribute to dysregulation of lipid and amino acid metabolisms in the meantime.

Metabolic changes in correlation with levels of immune reconstitution have rarely been explored. Current knowledge has demonstrated that INR status is partially linked to consistent immune activation despite long-term ART. Interestingly in our study, differences of identified metabolites in IRs and INRs were limited, with four significant metabolites in pre-treatment comparison and three in after-treatment, which was inconsistent with prior studies (6, 17). Shi Qian et al. found myristoylcarnitine (MC), palmitoylcarnitine (PC), stearoylcarnitine (SC), and oleoylcarnitine (OC) were significant elevated in INRs. Another research study found that HDL cholesterol and larger sizes of low-density lipoprotein particles had a better immunological recovery after treatment (16). However, we found that the sample size of identified metabolites in these studies were smaller than 20, and their conclusions were not consistent and had unclear pathogenies. Since the baseline viral replication level and CD4+T cell count were strictly limited, the less identified metabolites in these two groups may be more reliable. The identified metabolites in IRs and INRs in our study cover up Maltotetraose, N, N-dimethyl-5-aminovalerate, and decadienedioic acid (C10:2-DC). Maltotetraose is a normal human oligosaccharide, and it was elevated in cases of immune disease (33), mostly related to nutrition competition of microbiomes (34, 35). Further, N and N-dimethyl-5-aminovalerate were associated with catabolism of cadaverine from microbes (36), while Decadienedioic acid (C10:2-DC) belongs to the class of organic compounds known as medium-chain fatty acids (37). However, our study was explorative and not designed to establish causality, which lacks evidence of their biogenic effects and potential mechanism.

The current study has several limitations. Firstly, untargeted metabolomics analysis only provided the relative abundance of detected metabolites, so further quantitative studies are required to confirm the changes of the identified metabolites in the metabolomics study. Secondly, the study was retrospective, so the sample collection procedures and ART treatment might not be well controlled, which may result in nonspecific variations in the metabolomics study results. Other factors such as the duration of HIV infection, smoking habits, nutrient uptake, digestion status, timing of sampling, and co-infection status were not fully considered in the study, either. However, we collected their information about glucose, cholesterol, and blood pressure and found that our patients have similar metabolic levels. It is helpful to compensate for our limitations.

Overall, combining metabolomics analysis and cytokines measurement, we described the plasma metabolic-immune network of HIV patients before and after treatment in comparison with the healthy controls. The results indicated the central role of lipid and nucleotide metabolism disturbance in HIV infection and found that these metabolic changes could not be fully restored by viral suppressive ART. This was an explosive study and the underlying biological mechanisms of the metabolites described in the study require further validation.

## Data availability statement

The original contributions presented in the study are included in the article/**Supplementary Material**. Further inquiries can be directed to the corresponding authors.

## Ethics statement

The studies involving humans were approved by ethics committee and the institutional review board of PUMCH (Peking Union Medical College Hospital). The studies were conducted in accordance with the local legislation and institutional requirements. The participants provided their written informed consent to participate in this study.

## Author contributions

LL: Data curation, Formal Analysis, Writing – original draft. YY: Data curation, Writing – original draft, Writing – review & editing. ZY: Investigation, Resources, Software, Validation, Visualization, Writing – review & editing. YW: Data curation, Formal Analysis, Writing – review & editing. XL: Methodology, Resources, Software, Writing – review & editing. XL: Methodology, Resources, Software, Writing – review & editing. LC: Data curation, Resources, Software, Writing – review & editing. YH: Methodology, Resources, Supervision, Writing – review & editing. XS: Data curation, Project administration, Writing – review & editing. ZK: Funding acquisition, Methodology, Project administration, Supervision, Validation, Visualization, Writing – review & editing. WC: Conceptualization, Project administration, Resources, Supervision, Validation, Writing – review & editing. TL: Conceptualization, Funding acquisition, Project administration, Resources, Supervision, Writing – review & editing.

## Glossary

**Table:**

HIV-1	Human immunodeficiency virus type 1
ART	Antiretroviral treatment
PLWH	People living with HIV
INRs	Immunological non-responders
IRs	Immunological responders
OXPHOS	Oxidative phosphorylation
HCs	Healthy controls
UPLC-MS/MS	Ultra-high performance liquid chromatography-tandem mass spectrometry
CAWG	Chemical Analysis Working Group
MSI	Metabolomics Standards Initiative
IL	Interleukin
TNF	Tumor necrosis factor
CXCL-10	Chemokineligand-10
MCP-1	Monocyte chemotactic protein-1
ICAM-1	Intercellular Cell Adhesion Molecule-1
VCAM-1	Vascular Cell Adhesion Molecule-1
IFN	Interferon
CD40 ligand	CD40L
PCA	Principal component analysis
PLS-DA	Partial Least Square Discrimination Analysis
RF	Random Forest
VIP	Variable importance in the projection
FDR	False discovery rate
TCA	Tricarboxylic acid
IP-10	Interferon γ-induced protein 10 kDa
NRTIs	Nucleoside reverse-transcriptase inhibitors
NNRTI	Non-Nucleoside reverse-transcriptase inhibitors
PI	Proteinase inhibitor
INSTI	Integrase inhibitor
MC	myristoylcarnitine
PC	palmitoylcarnitine
SC	stearoylcarnitine
OC	oleoylcarnitine
IMP	inosine 5’-monophosphate
PUMCH	Peking Union Medical College Hospital
UMP	uridylic acid
ADP	Adenosine diphosphate
HDL	High density lipoprotein
