# Supplementary material for: Altered plasma metabolites and inflammatory networks in HIV-1 infected patients with different immunological responses after long-term antiretroviral therapy
Source: Front Immunol. 2023 Sep 27;14:1254155. doi: 10.3389/fimmu.2023.1254155 (PMC10565217; doi:10.3389/fimmu.2023.1254155)
Supplement: Supplementary file 1 [file DataSheet_1.docx]

Supplementary Figures：


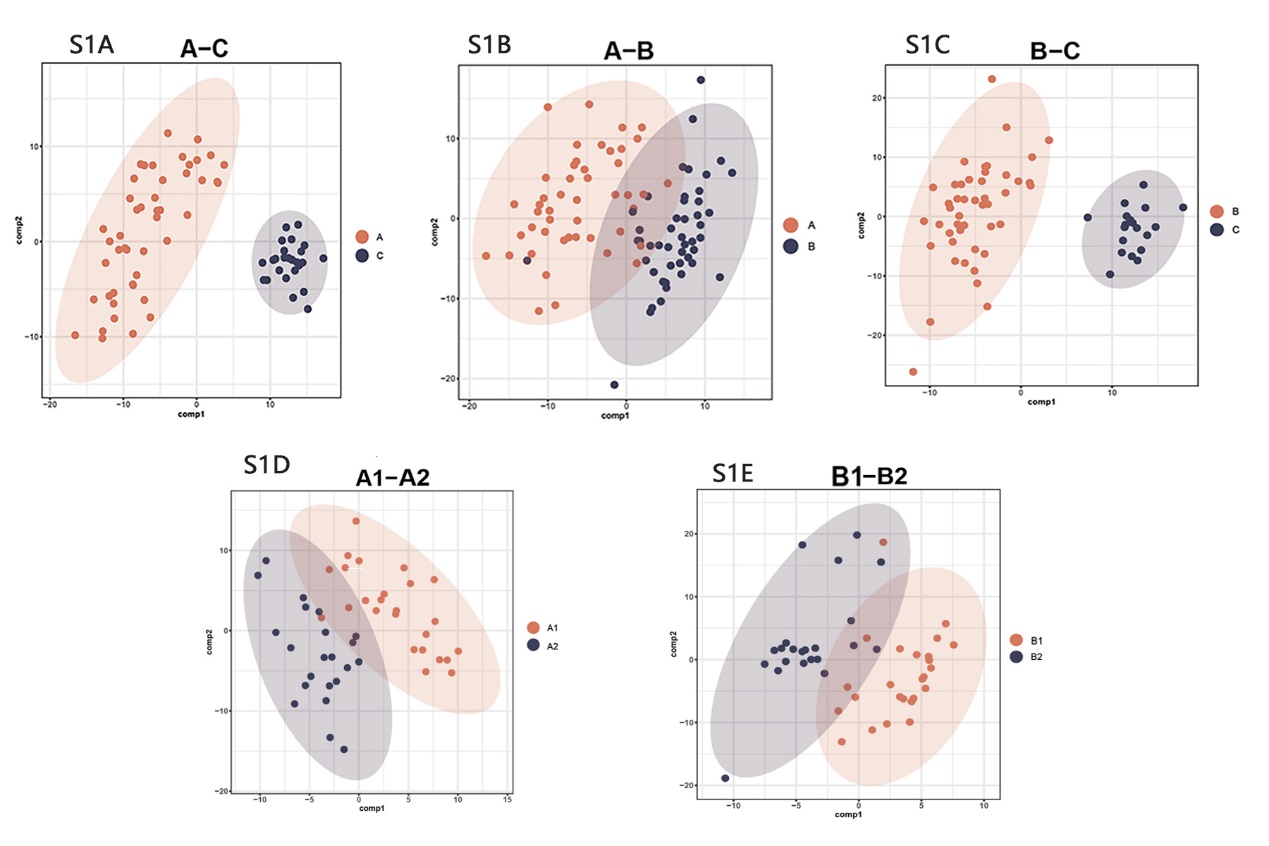


FigureS1: Partial Least Square Discrimination Analysis(PLS-DA) score plots derived from untargeted metabolism among different groups comparison: (S1a) PLS-DA plot identified group of HIV-infected patients prior to antiretroviral treatment(A) and healthy controls(C);(S1b) HIV-infected patients prior to antiretroviral treatment (A) and after ART treatment(B); (S1c) HIV-infected patients after ART treatment(B) and healthy controls(C); (S1d) HIV immune non-responders(A1) and immune responders(A2) prior to antiretroviral treatment. (S1e) immune non-responders(B1) and immune responders(B2) after antiretroviral treatment.


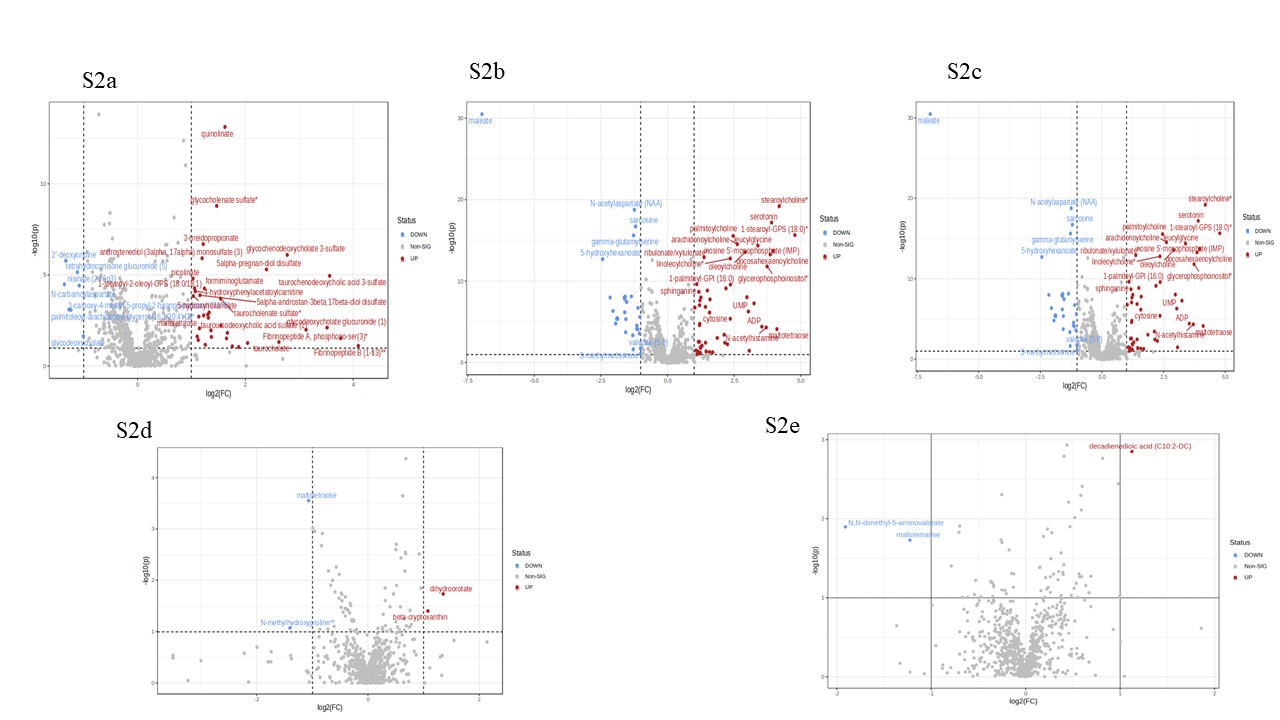


Figure S2: Volcano plots derived from untargeted metabolism among different groups comparison with lg (fold change)>1 and Holm. Adjusted p<0.1: (S2a) comparison of HIV-infected patients prior to antiretroviral treatment(A) and healthy controls(C);(S2b) HIV-infected patients prior to antiretroviral treatment (A) and after ART treatment(B); (S2c) HIV-infected patients after ART treatment(B) and healthy controls(C); (S2d) HIV immune non-responders(A1) and immune responders(A2) prior to antiretroviral treatment. (S2e) immune non-responders(B1) and immune responders(B2) after antiretroviral treatment.


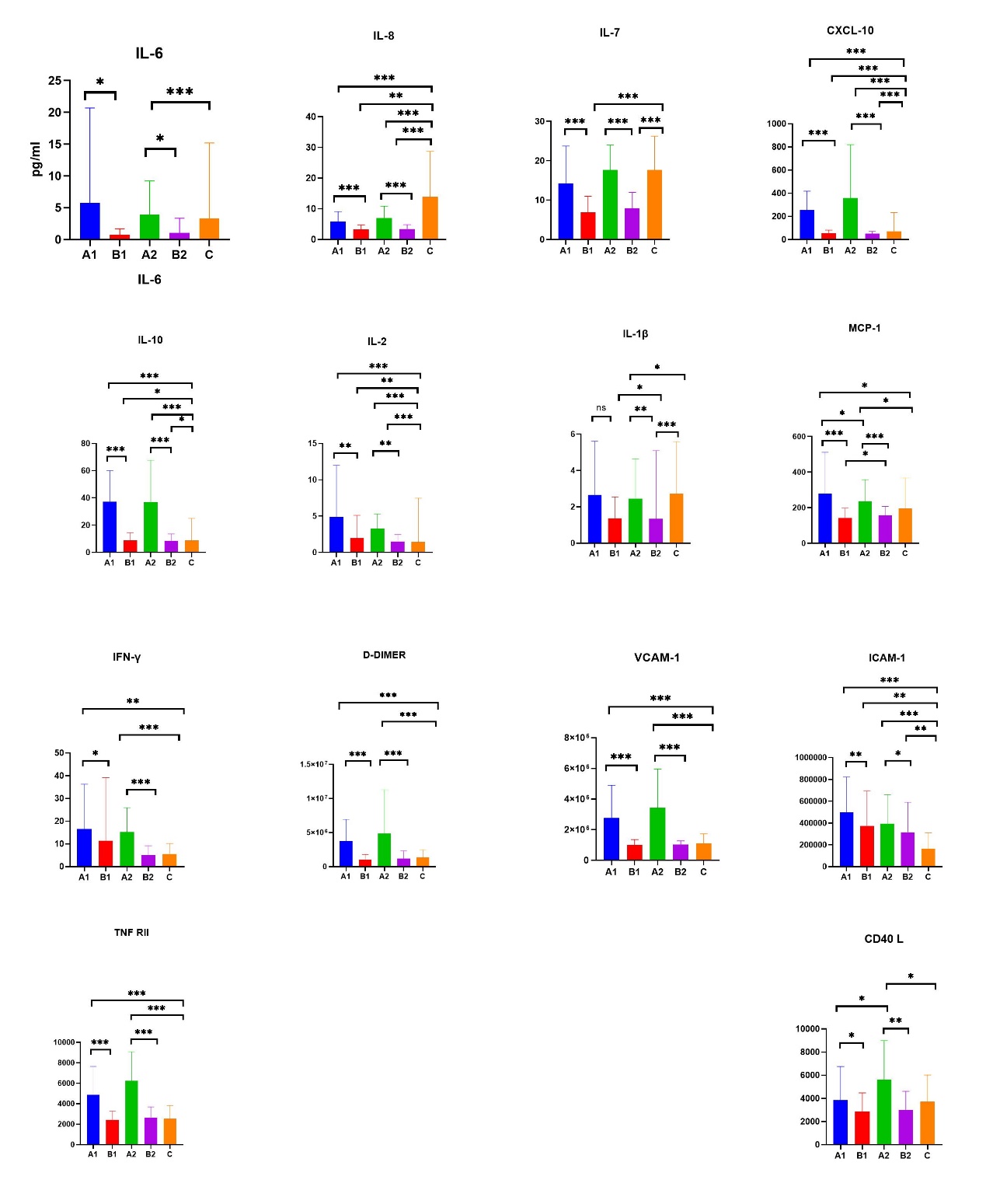


Figure S3: Box-and-whisker plots of the Plasma cytokine levels in A1 (n=25), A2(n=25), and B1(n=25), B2 (n=25) and C(n=25) samples. Mann Whitney test was used to compare every two of groups. *p<0.05, **p<0.01, ***p<0.001.

Supplementary Tables：

Table S1. Clinical characteristics of patients

| **Characteristics** | **Immunological non-responders**  **CD4 ≤350**  **N =25** | **Immunological responders**  **CD4 ≥ 500**  **N =25** | **Healthy Controls**  **N =25** | ***p*-value** |
| --- | --- | --- | --- | --- |
| Age, mean years (SD) | 45.8±11.7 | 43.3±10.3 | 44.7±12.4 | 0.554 |
| Male, n (%) | 24 (96.0) | 24 (96.0) | 23 (92.0) | 0.705 |
| Route of transmission, n (%)  Sexual  Blood  Unclear/others | 20 (80)  2 (8.0)  3 (12.0) | 19 (76.0)  2 (8.0)  4 (16.0) | - | 0.919 |
| Initial ART regimen, (%)  2 NRTIs + NNRTI  2NRTIs+PI  2 NRTIs + INSTI  Other | 20 (80.0)  1 (4.0)  4 (16.0)  0 (0.0) | 21 (84.0)  2 (8.0)  1 (4.0)  1 (4.0) | - | 0.368 |
| Switching ART regimen, n (%)  2 NRTIs + NNRTI  2NRTIs+PI  2 NRTIs + INSTI  Other | 18 (72.0)  4 (16.0)  3 (12.0)  0 (0.0) | 15 (60.0)  4 (16.0)  4 (16.0)  2 (8.0) | - | 0.491 |
| Viral load (lg copies/mL) | 5.07±0.73 | 5.13±0.61 | - | 0.752 |
| Weight(kg) | 59.8±10.7 | 66.0±10.0 | - | 0.042 |
| Baseline CD3+CD4+T counts (cells/ul) | 25 (7, 77) | 44 (13, 53) | - | 0.676 |
| Peak CD3+CD4+T counts (cells/ul) | 244 (201, 277) | 610 (555, 820) | - | <0.001 |

Notes: INRs: Immunological non-responders; IRs: Immunological responders; HCs: Healthy controls; SD: Standard deviation; NRTIs: Nucleoside reverse-transcriptase inhibitors; NNRTIs: Non-Nucleoside reverse-transcriptase inhibitors; PI: Proteinase inhibitor; INSTIs: Integrase inhibitor. “–” denotes no relevant data
